# The Prevalence of Self-Reported Diabetes in the Australian National Eye Health Survey

**DOI:** 10.1371/journal.pone.0169211

**Published:** 2017-01-03

**Authors:** Stuart Keel, Joshua Foreman, Jing Xie, Peter van Wijngaarden, Hugh R. Taylor, Mohamed Dirani

**Affiliations:** 1 Centre for Eye Research Australia, Royal Victorian Eye & Ear Hospital, Melbourne, Australia; 2 Ophthalmology, University of Melbourne, Department of Surgery, Melbourne, Australia; 3 Indigenous Eye Health Unit, Melbourne School of Population and Global Health, The University of Melbourne, Melbourne, Australia; Swinburne University of Technology, AUSTRALIA

## Abstract

**Objective:**

To present the prevalence of self-reported diabetes in Indigenous and non-Indigenous participants in the National Eye Health Survey.

**Research Design and Methods:**

3098 non-Indigenous Australians aged 50–98 years and 1738 Indigenous Australians aged 40–92 years were examined in 30 randomly selected sites, stratified by remoteness. A history of diabetes was obtained using an interviewer-administered questionnaire.

**Results:**

13.91% (431/3098) of non-Indigenous Australians and 37.11% (645/1738) of Indigenous Australians had self-reported diabetes. The age-adjusted prevalence of self-reported diabetes for non-Indigenous and Indigenous Australians was 11.49% and 43.77%, respectively (p <0.001). The prevalence of self-reported diabetes increased markedly with age (OR = 1.04 per year, p = 0.017). Indigenous Australians living in very remote areas were more likely to have self-reported diabetes than those in major city areas (OR = 1.61, p = 0.038).

**Conclusions:**

The prevalence of self-reported diabetes in Australia was high, with the prevalence being almost 4 times higher in Indigenous Australians compared with non-Indigenous Australians. With the prevalence of diabetes likely to increase, the results of this national survey may inform future policy, planning and funding allocation to assist in controlling the diabetes epidemic.

## Introduction

Diabetes and its complications are major causes of early death worldwide [1, 2]. Four hundred and fifteen million people worldwide (8.8% of adults aged 20–79 years) are estimated to have diabetes [3, 4]. This figure is expected to increase by at least 25% by 2030 to 552 million. This upward projection can be explained by population growth, an ageing population and a rise in the prevalence of risk factors for diabetes, including obesity [5]. In Australia alone, 1.5 million adults have diabetes and the number is predicted to increase to 2.9 million by the year 2025 [6, 7]. The prevalence of diabetes is much higher in Indigenous Australians and the onset of diabetes occurs at a significantly younger age than in non-Indigenous Australians [8–10].

The National Health Surveys (NHS) reported an increase in the prevalence of diabetes in Australia from 3% in those aged over 25 years and 7.5% in those aged over 65 years in 1989–90, to 5.1% and up to 13.2% in 2014–2015 in the same age groups [11, 12]. The landmark cohort study, the Australian Diabetes, Obesity and Lifestyle (AusDiab), reported an increase in the prevalence of clinically diagnosed diabetes in Australians aged 25 years or older from 8.5% at baseline (year 1999–2000), to 9.3% in 2004–2005 and to 12% in 2011–2012 [13–15]. The AusDiab remains the primary reference study for historical trends and projected forecasts for the prevalence of diabetes in Australian adults [16]. However, since the final wave of the AusDiab study in 2011–12 [15], there has been a rise in the prevalence of diabetes-related risk factors [17]. Furthermore, Indigenous Australians were underrepresented in these studies. Accordingly, more current population-based prevalence data that stratifies study participants by Indigenous status will prove valuable for monitoring the nationwide burden of diabetes.

Several studies have estimated the prevalence of diabetes in Indigenous Australians in a limited number of geographic areas [8, 9, 18–21] and only a few studies have reported the national prevalence of diabetes in this group [10, 22, 23]. The National Indigenous Eye Health Survey (NIEHS) was conducted in 2008 and found the prevalence of self-reported diabetes in Indigenous Australians age 40 years and over to be 37.3%, approximately 8 times higher than that of the general population of Australia [24]. The most recent Australian Aboriginal and Torres Strait Islander Health Survey (2012–13) found that approximately one in six (18%) Indigenous persons aged 25 years and over had self-reported diabetes, with the prevalence increasing to 39% in those aged 55 years or more [23]. Considering the burden of diabetes in Indigenous Australians, up-to-date prevalence data in this high-risk group through a population-based survey is required.

This paper presents the prevalence of self-reported diabetes in Indigenous and non-Indigenous Australians recruited from 30 different regions in Australia as part of the National Eye Health Survey (NEHS).

## Materials and Methods

### Study design and response rates

The NEHS was a cross-sectional, nationwide population-based study conducted from the 11th of March 2015 to the 18^th^ of April 2016. Using a multi-stage, random cluster sampling methodology, 30 geographic areas stratified by remoteness were randomly selected to provide a representative sample of non-Indigenous and Indigenous Australian adults. Participants were deemed eligible to participate if they were non-Indigenous and aged 50 years or older or Indigenous and aged 40 years or older, non-institutionalised and were living at the residence at the time of recruitment. Sites were selected from a sampling pool of Statistical Areas (SAs) using the Australian Statistical Geography Standard (ASGS) developed by the Australian Bureau of Statistics (ABS) to report 2011 Census data [25]. SAs were selected from all levels of geographic remoteness, including major city, inner regional, outer regional, remote and very remote areas.

The primary recruitment methodology involved door-to-door household recruitment, however the recruitment of Indigenous Australians included some modifications to be culturally sensitive and to account for the diversity of local conditions. Alternative methods of recruitment included; telephone recruitment from community lists, word of mouth, media and public relations and recruitment from concurrent Aboriginal Health Service clinics. Collaboration with community elders and local health workers at each site facilitated community acceptance of the survey and assisted recruitment. A total of 11,883 residents were contacted across the 30 NEHS sites, of whom 6,760 (56.9%) were eligible to participate in the survey. Of those deemed to be eligible, a total of 5,764 agreed to participate, resulting in a positive response rate of 85.3% (5,764/6,760). Of these, 4,836 residents attended NEHS testing venues and underwent examinations, resulting in an overall clinical examination rate of 71.5% (4,836/6,760). The protocol for this study was approved by the Royal Victorian Eye and Ear Hospital Human Research Ethics Committee (HREC-14/1199H) and additional ethical approvals were obtained at the State level from the Aboriginal Health and Medical Research Council of NSW (HREC-1079/15), the Menzies School of Health Research (HREC-2015-2360), the Aboriginal Health Council of Western Australia (HREC-622) and the Aboriginal Health Council of South Australia (HREC-04-15-604) to conduct research within Indigenous communities. Participation was incentivised with a free pair of sunglasses. All participants provided written informed consent.

### Interviewer-administered questionnaire

Surveys took place in venues within 6 km of each targeted survey site. Each participant underwent an interviewer-administered questionnaire (S1 File). This questionnaire, developed by the research team, ascertained key socio-demographic data including date of birth, gender, level of education, country of birth, main language spoken at home and Indigenous/non-Indigenous status. Participants who identified as Indigenous were further classified as 1) Aboriginal, 2) Torres Strait Islander or 3) Aboriginal and Torres Strait Islander. Using questionnaire data, ethnicity was later categorised according to the Australian Standard Classification of Cultural and Ethnic Groups (ASCCEG) 2011 for non-Indigenous participants [26]. The component of the questionnaire pertaining to diabetes history asked participants whether they had ever been told by a doctor or nurse that they have diabetes (self-reported diabetes). Interviewers then ascertained the age of diagnosis in those with self-reported diabetes to determine the duration of disease.

### Statistical analysis

Participant demographic characteristics were summarised as the mean and standard deviation (SD) for normally distributed continuous data, or the median and inter-quartile range for skewed distributed data, and counts and percentages for categorical data. Key covariates included age (years), gender, education years, ethnicity, main language spoken at home (English/other), and remoteness. Due to small participant numbers in some of the ethnic group sub-categories, non-Indigenous participants were grouped into Oceanian (Australian Peoples, New Zealand Peoples, Melanesian and Papuan, Micronesian, Polynesian), European and other for analyses. Multivariable logistic regression analysis was used to examine the association between self-reported diabetes and all covariates that were significant in univariate analysis. For the final fitted logistic regression model, we examined model residuals and delta beta values to determine if potential outlying observations influenced analysis results, and we also assessed the degree to which statistical assumptions were violated. Associations were considered statistically significant if p<0.05. All statistical analyses were undertaken using Stata version 14.1 (StataCorp, College Station, TX).

## Results

### Prevalence of self-reported diabetes

A total of 4,836 individuals were recruited and examined in the NEHS, including 3,098 (64.06%) non-Indigenous Australians and 1,738 (35.94%) Indigenous Australians, respectively. Of these, 431 (13.91%, 95% CI: 12.71%-15.18%) non-Indigenous Australians (with mean [SD] duration of diabetes = 12.50 [10.0] years) had self-reported diabetes (age-adjusted prevalence = 11.49%). In contrast, Indigenous Australians (with mean [SD] duration of diabetes = 13.18 [10.86] years) had a crude prevalence of self-reported diabetes nearly three times higher (37.11%) than that of their non-Indigenous counterparts (p<0.001). The age-adjusted prevalence of self-reported diabetes was four times higher in Indigenous participants (43.77%) than in non-Indigenous participants (p<0.0001). When matched for age, Indigenous Australians were significantly younger at diabetes diagnosis when compared to non-Indigenous Australians (Indigenous mean [SD] age of diabetes onset = 47.41 [12.21] years vs. non-Indigenous = 56.10 [13.81] years, p = <0.001).

### Differences between those who self-reported diabetes versus those who did not

#### Non-Indigenous participants

Non-Indigenous participants who self-reported diabetes were significantly older (mean [SD] = 68.62 [8.90] years vs. 66.24 [9.77] years, p = <0.001) and had fewer years of educational attainment (mean [SD] = 12.06 [3.78] years vs. 12.61 [3.72], p = 0.004) than those who did not self-report diabetes (Table 1). The proportion of participants who were male differed significantly between those who had diabetes and those who did not, with 56.15% of diabetics being male, compared with 44.81% of non-diabetics being male (p<0.001). A smaller proportion of non-Indigenous participants with self-reported diabetes were found to speak English at home compared with their non-diabetic counterparts (88.86% vs. 95.24%, p = <0.001). The prevalence of self-reported diabetes increased with age until it plateaued after the age of 70 years (age 50–59 years, 8.85%; 60–69 years, 14.82%; 70–79 years, 16.14%; >80 years, 17.74%; p = <0.0001).

**Table 1:** Comparison of key demographics between participants with self-reported diabetes and those without, stratified by Indigenous status.

	Non-Indigenous			Indigenous		
	No diabetes (n = 2667)	Diabetes (n = 431)	p	No diabetes (n = 1093)	Diabetes (n = 645)	p
Age (year)	66.24 (9.77)	68.62 (8.90)	<0.001	53.07 (9.58)	58.31 (9.75)	<0.001
Education (year)	12.61 (3.72)	12.06 (3.78)	0.004	11.26 (3.28)	10.51 (3.31)	<0.001
Gender (male)	1195 (44.81)	242 (56.15)	<0.001	472 (43.18)	242 (37.52)	0.020
English spoken at home	2540 (95.24)	383 (88.86)	<0.001	1093 (97.44)	606 (93.95)	<0.001
Ethnicity						
Oceanian	1929 (72.33)	287 (66.59)	0.004			
European	558 (20.92)	97 (22.51)				
Others	180 (6.75)	47 (10.90)				

Statistical significance was set as a p value of <0.05 (two-tailed).

#### Indigenous participants

As was found in non-Indigenous participants, Indigenous participants who self-reported diabetes were significantly older (mean [SD] = 58.31 [9.58] years vs. 53.09 [9.58] years, p<0.001), had fewer years of educational attainment (mean [SD] = 10.51 [3.31] years vs. 11.26 [3.28], p<0.001) and had a lower proportion of English speakers (self-report diabetes = 93.95% vs. no diabetes = 97.44%, p<0.001) compared to those who did not self-report diabetes. The proportion of Indigenous Australians with diabetes who were male (37.52%) was significantly lower than the proportion who were male in the non-diabetic group (43.15%, p = 0.020). Similarly to non-Indigenous Australians, the prevalence of self-reported diabetes increases markedly with age, plateauing after the age of 70 years (age 40–49 years, 21.78%; 50–59 years, 37.50%; 60–69 years, 53.87%; >70 years, 53.42%; p = <0.0001).

### Associations between risk factors and self-reported diabetes

#### Total population

The univariate and adjusted logistic regression analysis revealed significant associations between self-reported diabetes and several socio-demographic variables in the total population (Table 2). After controlling for covariates, Indigenous status was the greatest risk factor for self-reported diabetes, with an odds ratio of 5.91 (95% CI: 4.86, 7.19). After adjustments, the prevalence of self-reported diabetes increased significantly with age (OR = 1.04 per year, 95% CI: 1.03, 1.05) and English speakers at home (OR = 0.46,95% CI: 0.33, 0.65) were at a lower risk of self-reported diabetes. Due to differences in the age inclusion criteria and sampling methodologies employed between Indigenous and non-Indigenous participants, further logistic regression analysis was stratified by Indigenous status.

**Table 2:** Univariate and multivariable logistic regression analysis of associations between self-reported diabetes and sociodemographic factors.

	All study participants (n = 4,836)				Non-Indigenous (n = 3,098)				Indigenous (n = 1,738)			
Associated factors	Univariate		Multivariate^2^		Univariate		Multivariate^4^		Univariate		Multivariate^6^	
	Unadjusted OR (95% CI)	p*	Adjusted OR (95% CI)	p	Unadjusted OR (95% CI)	p	Adjusted OR (95% CI)	p	Unadjusted OR (95% CI)	p	Adjusted OR (95% CI)	p
Indigenous	3.65 (3.17, 4.20)	<0.001	5.91 (4.86, 7.19)	<0.001								
Age (year)	1.00 (0.99, 1.01)	0.928	1.04 (1.03, 1.05)	<0.001	1.03 (1.02, 1.03)	<0.001	1.02 (1.01, 1.03)	<0.001	1.06 (1.04, 1.07)	<0.001	1.05 (1.04, 1.07)	<0.001
Gender (male)	1.03 (0.90, 1.18)	0.707	1.10 (0.95, 1.27)	0.215	1.58 (1.28, 1.94)	<0.001	1.61 (1.30, 1.98)	<0.001	0.79 (0.65, 0.96)	0.021	0.75 (0.60, 0.92)	0.007
Education (year)	0.92 (0.90, 0.94)^1^	<0.001	0.97 (0.95, 1.00)	0.017	0.96 (0.93, 0.99)^3^	0.007	0.97 (0.64, 0.99)	0.021	0.93 (0.90, 0.96)^5^	<0.001	0.98 (0.94, 1.01)	0.134
English at home	0.49 (0.37, 0.64)	<0.001	0.46 (0.33, 0.65)	<0.001	0.40 (0.28, 0.57)	<0.001	0.49 (0.32, 0.75)	0.001	0.41 (0.90, 0.96)	<0.001	0.45 (0.26, 0.76)	0.003
***Ethnicity***												
Oceanian	1		1		1		1					
European	0.58 (0.46, 0.72)	<0.001	0.97 (0.75, 1.27)	0.849	1.17 (0.91, 1.50)	0.220	1.01 (0.77, 1.31)	0.968				
Others	0.84 (0.61, 1.18)	0.327	1.42 (0.96, 2.12)	0.077	1.75 (1.24, 2.48)	0.001	1.45 (0.95, 2.20)	0.083				
***Remoteness***												
Major City	1		1		1		1		1		1	
Inner Regional	0.88 (0.72, 1.06)	0.171	0.93 (0.76, 1.15)	0.507	1.03 (0.79, 1.36)	0.807	1.10 (0.83, 1.45)	0.521	0.81 (0.61, 1.07)	0.150	0.80 (0.59, 1.09)	0.155
Outer Regional	1.19 (1.00, 1.42)	0.054	1.12 (0.93, 1.36)	0.230	1.06 (0.80, 1.39)	0.695	1.05 (0.80, 1.40)	0.716	1.28 (1.00, 1.64)	0.053	1.26 (0.97, 1.63)	0.083
Remote	0.80 (0.63, 1.01)	0.062	0.80 (0.62, 1.03)	0.080	0.72 (0.50, 1.04)	0.078	0.76 (0.52, 1.11)	0.154	0.95 (0.68, 1.34)	0.778	0.86 (0.59, 1.24)	0.411
Very Remote	1.12 (0.84, 1.48)	0.438	1.19 (0.89, 1.60)	0.235	0.89 (0.58, 1.37)	0.610	0.986 (0.62, 1.49)	0.867	1.78 (1.16, 2.73)	0.008	1.61 (1.03, 2.52)	0.038

OR = Odds Ratio

CI = Confidence Interval

* Statistical significance was set as a p value of ≤0.05 (two tailed).

^1^ 44 participants did not report how many educational years.

^2^ The likelihood ratio test of the model was statistically significant (n = 4792, χ2 = 495.20, p <0.001)

^3^ 14 participants did not report how many educational years

^4^ The likelihood ratio test of the model was statistically significant (n = 3084. χ2 = 84.94, p <0.001). Model was controlling for age, gender, ethnicity, education, English-speaking at home, ethnicity and remoteness.

^5^ 30 participants did not report how many educational years

^6^ The likelihood ratio test of the model was statistically significant (n = 1046, χ2 = 119.40, p <0.001). Model was controlling for age, gender, education, English-speaking at home, and remoteness

#### Stratified by Indigenous status

In the non-Indigenous population, adjusted analysis revealed that male gender (OR = 1.61, 95% CI: 1.30, 1.98), older age (OR = 1.02 per year, 95% CI: 1.01, 1.03) and lower number of years of education (OR = 0.97 per year, 95% CI: 0.64, 0.99) were risk factors for self-reported diabetes, while English speakers at home (OR = 0.49, 95% CI: 0.32, 0.75) were at lower risk for self-reported diabetes.

For Indigenous Australians, multivariate analysis revealed that older age (OR = 1.05, 95% CI: 1.04, 1.07), female gender (OR = 0.75, 95% CI: 0.60, 0.92) and residing in very remote geographical areas (1.61, 95% CI: 1.03, 2.52) were significant risk factors for self-reported diabetes, while those who spoke English at home (OR = 0.45, p = 0.003) were at lower risk of self-reported diabetes.

## Discussion and Conclusion

This paper presents the prevalence of self-reported diabetes in Indigenous and non-Indigenous Australian participants in a cross-sectional study from 30 randomly selected sites across the nation. The age-adjusted prevalence of self-reported diabetes was almost 4 times higher in Indigenous participants (43.77%) than in their non-Indigenous counterparts (11.49%). There was a clear association between increasing age and increased diabetes prevalence (OR = 1.02 per year of age, and 1.05 per year of age, for non-Indigenous and Indigenous participants, respectively, p< 0.001).

In 2008, it was estimated that the prevalence of diabetes would rise to 11.5% for those aged 45–64 years and 19.8% for those aged 65–85 years by the year 2016 [14, 27]. Our findings are closely aligned with these projections. The increasing prevalence of diabetes in Australia has been indicated by the National Health Survey (8.9–10.9%, 55–64 years) [11] and by the AusDiab study (9.4%, 40–74 years) [13]. It must be noted that estimates from the AusDiab were obtained using plasma glucose measurements and the diagnosis of diabetes for approximately half of participants was made during the course of the study. Therefore, the use of self-reported diabetes in the present study will have underestimated the true prevalence of disease. The prevalence of self-reported diabetes identified in the present study is consistent with estimates made by statistical modeling [16, 5]. Likely risk factors associated with the increase in diabetes prevalence include an ageing population, rising obesity rates and population growth [6]. Of note, our finding that non-English speaking participants were at higher risk of diabetes is consistent with previous Australian reports [28]. Genetics, immigration stressors, socio-economic and cultural factors have been implicated in the higher level of diabetes risk in this Australian sub-group [29]. Our data highlights that more work is required to address the modifiable barriers to the effective and equitable delivery of health services, including; language barriers, literacy rates, effects of stigmatisation and lack of access to culturally specific care [30].

Our findings are consistent with previous epidemiological evidence reporting a significantly higher prevalence of diabetes in Indigenous Australians compared to non-Indigenous Australians, and with an earlier age of onset of the disease [8–10]. We found that the prevalence of self-reported diabetes (43.77%) was higher than that identified in the NIEHS in 2008 (37.3%) [10]. Given the similarities in the study methods and examination rates for Indigenous participants (NEHS = 77.6% vs. NIEHS = 78.7%), robust comparisons can be made between the findings of these studies. Accordingly it appears that the prevalence of diabetes in Indigenous Australians is on an upward trajectory. These findings are supported by those of the Australian Aboriginal and Torres Strait Islander Health Survey, that reported an increase in the prevalence of diabetes in similarly aged participants (>55 years) from 32% in 2005 to 39% in 2013 [22, 23]. Previous evidence has implicated a greater genetic predisposition [31] and a rising prevalence of key diabetes risk factors, such as obesity [8, 23, 32], poor nutrition [23, 33] and low physical activity levels [23], in the higher prevalence of diabetes in Indigenous Australians. The recent Aboriginal and Torres Strait Islander Health Survey (2012–13) highlighted this disparity, reporting that Indigenous Australians displayed 1.5 times higher rates of obesity, were approximately half as likely to meet national physical activity targets and exhibited significantly poorer adherence to recommended fruit and vegetable intake guidelines when compared to non-Indigenous Australians [23]. Efforts to address these issues through the implementation of primary prevention strategies are ongoing [34].

The higher prevalence of diabetes observed in Indigenous Australians living in very remote regions concurs with findings reported by Hoy and co-workers (2007) who found that the prevalence of diabetes in Indigenous persons from three remote communities were 5.4-10-fold higher than that of the general population [9]. These findings may be related to multiple barriers that Indigenous Australians face in accessing specialist services in very remote areas, including communication, distance, and cultural factors [28].

This is the first study of its kind to provide population-based national data on the prevalence of self-reported diabetes in both non-Indigenous and Indigenous Australian adults. Key strengths of this study include the high positive response and examination rates and a representative nationwide population sample. The primary limitation of this study was the use of self-report for the ascertainment of diabetes. While we cannot deny the potential influences of self-reporting bias, self-report is a commonly utilised tool for diabetes surveillance [10, 12, 30, 31]. In addition, several studies have reported high sensitivity and specificity for diabetes self-reporting as an indicator of medically diagnosed diabetes [32–34]. The absence of glycemic testing in the study protocol is likely to have resulted in an underestimation of the true prevalence of diabetes, as it is estimated that up to 50% of cases are undiagnosed [13].

The prevalence of self-reported diabetes in Australia is high and is increasing. Notably, the age-adjusted prevalence of diabetes in Indigenous Australians in this study was almost 4 times higher than in non-Indigenous Australians. These findings portend major public health and economic challenges for Australia.

## Supporting Information

S1 File General questionnaire. (DOCX)
