# Supplementary material for: The Prevalence of Self-Reported Diabetes in the Australian National Eye Health Survey
Source: PLoS One. 2017 Jan 3;12(1):e0169211. doi: 10.1371/journal.pone.0169211 (PMC5207759; doi:10.1371/journal.pone.0169211)
Supplement: S1 File — (DOCX) [file pone.0169211.s001.docx]

**General questionnaire**

**National Eye Health Survey questionnaire (hard copy)**

Interviewer Initials: _ _ _

Interviewer Code: NEHS _ _

Date of Examination: _ _ (dd) /_ _ (mm) /_ _ _ _ (yyyy)

Time of Examination: _____ am/pm

Participant Unique ID: NEHS _ _ _ _

1. **Personal Particulars:**
   1. What is your given name?__________________________________________
   2. What is your surname?____________________________________________
   3. What is your gender? Tick the correct option

Male

Female

d. What is your age? _______

e. What is your date of birth? _ _ (dd) /_ _ (mm) /_ _ _ _ (yyyy)

1. **Ethnicity**:
   1. What is your country of birth? Tick the correct option

Australia

England

New Zealand

China

India

Italy

Vietnam

Philippines

South Africa

Other, please specify___________________________________________

- 1. If Australia was not your place of birth, how many years have you been in Australia? Record as a whole number
     __________________________________________________________
  2. Are you of Aboriginal or Torres Strait Islander origin? Tick the correct option

Yes, Aboriginal

Yes, Torres Strait Islander

Yes, Aboriginal and Torres Strait Islander

No

- 1. What is the main language you speak at home? Tick the correct option

English

Italian

Greek

Cantonese

Arabic

Vietnamese

Indigenous language, please specify_______________________________

Other, please specify___________________________________________

1. **Educational Attainment:**
2. What is your highest level of education? Tick the correct option

    Grade 0 = No education
    Grade 1 = Primary education incomplete
    Grade 2 = Completed primary education
    Grade 3 = Completed primary and some years of secondary education
    Grade 4 = Completed primary and secondary education
    Grade 5 = Attending/completing trade school or TAFE
    Grade 6 = University student
    Grade 7 = Completed university degree
    Grade 8 = Undertaking/completed post graduate study
3. Total number of years of education _____ years Record in years
4. **Stroke:**
   1. Have you ever had a stroke? Tick the correct option

Yes

No

1. **Past Ocular History:**
   1. Have you ever had your eyes examined? Tick the correct option

Yes

No (Proceed to d)

- 1. If yes, how long ago? Record in years and months _____ years _____ months
  2. Who did you see for your eye examination? Tick more than one

Optometrist

Eye Doctor/Ophthalmologist

GP/Local Doctor

Nurse

Health Worker

Ophthalmic Nurse/Technician

Other, please specify___________________________________________

- 1. Have you ever been told that you have any of the following eye conditions?

Tick the correct option for **each** eye condition.

|  |  |  |
| --- | --- | --- |
|  |  |  |
|  |  |  |
|  |  |  |

|  |  |  |
| --- | --- | --- |
|  |  |  |
|  |  |  |

| **Eye Condition** | **Yes** | **No** | **Unsure** |
| --- | --- | --- | --- |
| Glaucoma (high pressure in the eye) |  |  |  |
| Diabetic Retinopathy (diabetic eye disease) |  |  |  |
| Age-related macular degeneration (loss of your central vision) |  |  |  |
| Refractive error (wear glasses) |  |  |  |
| Cataracts (cloudiness of the lens resulting in decreased vision) |  |  |  |

Other, please specify: _______________________________________________________________

- 1. Have you ever had cataract surgery? Tick the correct option

Yes

No (Proceed to Question 6)

- 1. If yes, to which eye? Tick the correct option

Right

Left

Both eyes

- 1. If yes, how long ago (please specify for each eye)?Record in years and months

R) _____ years _____ months L) _____ years _____ months

1. **Diabetes and Duration:**
   1. Have you been told by a doctor or nurse that you have diabetes? Tick the correct option.

Yes
 No (Proceed to Question 7)

- 1. If yes, at what age were you first told that you had diabetes? Record in years
     _____ years old
  2. Have you seen an Eye Doctor/Ophthalmologist or Optometrist for a diabetes eye check? Tick the correct option

Yes

No (Proceed to e)

- 1. If yes, how long ago? Record in years and months
     _____ years _____ months

e. If no, why? Tick the correct option
 I did not know
 I was not told
 I missed the appointment
 I have no time
 Other, please specify____________________________________________

1. **Refractive error:**
   1. Do you wear the following? Tick more than one

Glasses

Contact lenses

I currently do not wear glasses or contact lenses (Questionnaire complete)

- 1. If you do wear glasses or contact lenses, are they for: Tick the correct option

Distance (driving or watching TV)

Near (reading or computer work)

Both

c. At what age did you first wear glasses? Record in years

_____ years old
